# Activity of the Upstream Component of Tandem TERT/Survivin Promoters Depends on Features of the Downstream Component

**DOI:** 10.1371/journal.pone.0046474

**Published:** 2012-10-03

**Authors:** Irina V. Alekseenko, Victor V. Pleshkan, Eugene P. Kopantzev, Elena A. Stukacheva, Igor P. Chernov, Tatyana V. Vinogradova, Eugene D. Sverdlov

**Affiliations:** 1 Institute of Molecular Genetics, Russian Academy of Sciences, Moscow, Russia; 2 Shemyakin and Ovchinnikov Institute of Bioorganic Chemistry, Russian Academy of Sciences, Moscow, Russia; University of Massachusetts Medical, United States of America

## Abstract

We spliced the promoters of the human telomerase and human survivin genes (PhTERT and PhSurv, respectively) widely used for gene therapy and known to have the broadest cancer type spectrum of activity. Two head-to-tail constructs were obtained: the PhTERT-PhSurv and PhSurv-PhTERT tandems. The splicing caused quantitative and qualitative changes in the promoter features. In both constructs, only the promoter proximal to the transcribed gene retained its ability to initiate transcription, whereas the distal promoter was silent, the phenomenon never reported before. However, the distal promoter modulated the activity of the proximal one by increasing its strength and causing an appearance of additional transcription start sites. We suggested that this suppression might be due to the presence of Sp1 transcription factor binding sites in both promoters and Sp1-bridges between these sites. Such Sp1-bridges might convert the tandem promoter linear DNA into a stem-loop structure. If localized inside the formed loop, the distal promoter could lose its ability to initiate transcription. To test this hypothesis, we constructed two modified double promoters, where the proximal PhSurv promoter was replaced either by a shortened variant of the survivin promoter (PhSurv269) or by the mouse survivin promoter. Both PhSurv substitutes were considerably shorter than PhSurv and had different numbers and/or positions of Sp1 sites. In modified tandems, transcription was initiated from both promoters. We also prepared two mutant forms of the PhSurv-PhTERT tandem with two or four Sp1 sites removed from the distal “long” PhSurv promoter. In the first case, the distal PhSurv promoter remained silent, whereas the removal of four Sp1 binding sites restored its activity. In the majority of studied cancer cell lines the efficiency of transcription from the hTERT-(shortened hSurv269) promoter tandem was markedly higher than from each constituent promoter. In normal lung fibroblast cells, the tandem promoter activity was considerably lower.

## Introduction

Gene therapy represents treatment modality that offers unique opportunities for tumor targeting. To this end, cellular mechanisms of gene regulation have been successfully used to direct therapeutic gene expression preferentially to cancer cells [1]. This approach called transcriptional targeting exploits cellular gene regulatory elements that mediate cell type-specific transcription to restrict the expression of therapeutic genes to only cancer cells. To be efficient, this system should provide sufficiently strong and specific expression of the transgene. Usually, natural tissue- or tumor-specific promoters are used for this purpose. Among them, there are, in particular, the promoters of the cyclooxygenase-2 (Cox-2), telomerase reverse transcriptase (hTERT), carcinoembryonic antigen (CEA), serum alpha-feto-protein (AFP), and prostate-specific antigen (PSA) genes, as well as the promoter (PhSurv) of the BIRC5 (survivin) gene of the apoptosis inhibitor survivin [2], [3], [4], [5]. The list of such promoters is continuously expanding. However, they have two important drawbacks. First, they are relatively weak as compared with, for example, the strong constitutive CMV or SV40 promoters. Second, most of the promoters described are active only in a few cancer cell types [2], [4]. A perfect universal cancer-specific promoter should work in many different tumors but not in normal cells. Moreover, it should successfully work not only in the primary tumor, but also in its metastases. At present, the PhSurv promoter directing transcription of the BIRC5 (survivin) gene and the PhTERT promoter directing transcription of the telomerase catalytic subunit gene (*hTERT*) are considered to be close to these requirements and widely used for gene therapy purposes. Although both promoters have a rather broad activity spectrum, they are still very far from being universal: e.g. PhSurv and PhTERT are active in tumors of only about 60% of patients with non-small cell lung cancer [6], [7]. In addition, the relative activity of these promoters significantly varies in different tumor cell lines [3], [8], [9].

Our preliminary data (to be published) showed that the activity profiles of the two constituent promoters were to a great extent complementary with partial overlapping. Therefore, we hypothesized that a tandem combination of the PhSurv and PhTERT promoters might represent a more universal and strong promoter.

Tumor-specific double (tandem) promoters were used in recent works [10], [11]. Using promoters of the *hASH1* and *EZH2* genes, Poulsen at al. constructed a chimeric double promoter for efficient expression of a killer gene in cells of small cell lung cancer [10]. The activity of the double promoter 2–8 fold exceeded that of the constituent single promoters, depending on the cell line tested.

High-level expression of the tBid apoptosis activator in cells of breast cancer was achieved with a hybrid promoter constituted of PhSurv and the promoter of a gene coding for mucin and known to be upregulated in tumor cells of the mammary gland [11].

In both cases above [10], [11], the authors aimed to create a promoter highly active only in specific types of cancer. In this study, we, to our knowledge, for the first time, made an attempt to construct double head-to-tail organized promoters PhTERT-PhSurv and PhSurv-PhTERT (hereafter referred to as PhTS and PhST, respectively) in order to obtain a universal cancer specific promoter. We assessed the efficiency of constructed tandems PhTS and PhST in driving the expression of a reporter gene in tumor cells as compared to the single constituent promoters. The tandems were constructed from a 1.5 kb survivin promoter (PhSurv) and the minimal hTERT promoter (PhTERT). We also determined the location of transcription start sites (TSSs) in single and double promoters under study. The properties of both tandem promoters were found to be strikingly different from additive properties of their constituents. Unexpectedly, we revealed a new type of promoter interference phenomenon, due to which the distal promoter activity in the tandems was suppressed, and transcription was initiated only at the proximal promoter. However, the distal promoter modulated the activity of the proximal one by increasing its strength and causing an appearance of additional transcription start sites. The number of TSSs in the proximal promoters was considerably increased compared with that in the same individual promoters. The suppression of the distal promoter was observed both in the PhTS and PhST promoters. However, this effect disappeared when the proximal promoter of PhTS was replaced with either a shorter version of PhSurv (PhSurv269) or by the mouse survivin promoter. Moreover, the effect of the distal promoter suppression disappeared in the PhSurv-PhTERT tandem when four Sp1 sites of the PhSurv constituent were functionally inactivated by mutations.

This report is mainly focused on deciphering the mechanism of interference leading to inactivation of distal promoters in tandems and the ways to avoid this effect when designing tandem promoters for different purposes. However, it is worthy of attention that one of the investigated promoters, PhTERT-PhSurv269, was significantly more active than others. In the majority of studied cancer cell lines, the initiation level of transcription from this tandem was on the average 4.8 and 3.3 times higher than that of the PhTERT and PhSurv cancer-specific promoters, respectively. In normal lung fibroblast cells, the tandem promoter activity was considerably lower.

## Results and Discussion

### Characteristics of promoters used in the study

**Figure 1:**
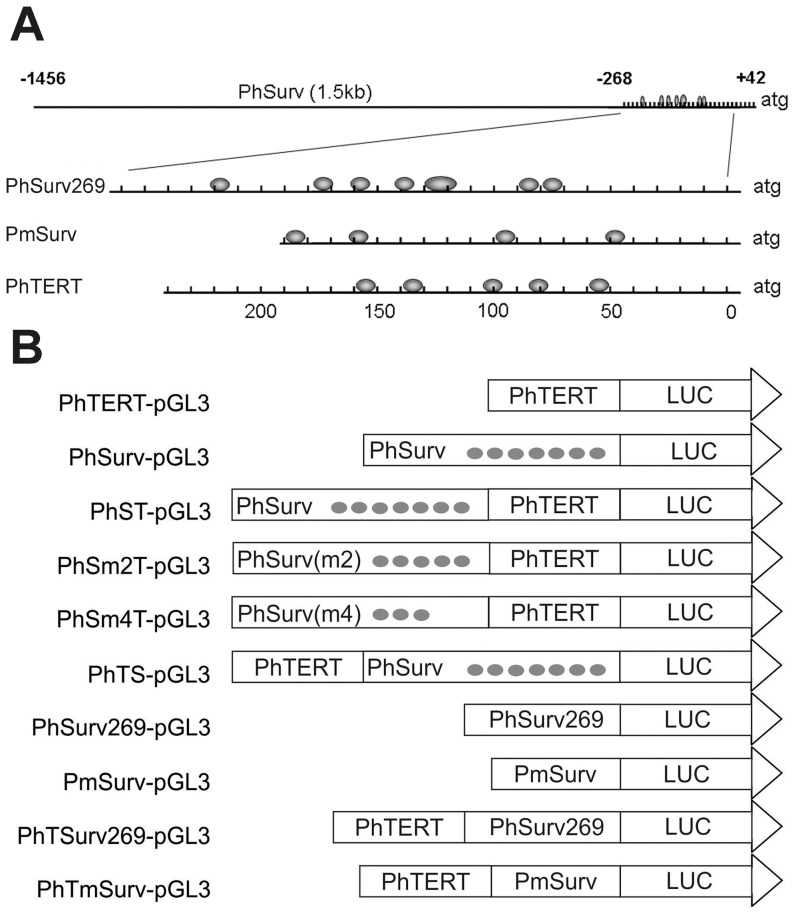
Structure of the promoters used in the study. **A:** Schematic representation of the Sp1 binding sites in the promoters under study. Gray ellipses denote Sp1 binding sites determined previously [12], [16], [17]. The scale is graduated in base pairs; atg denotes the relative position of the firefly luciferase gene start codon. **B:** Schematic representation of the expression constructs used. Gray circles denote active Sp1 sites in the intact and mutant promoters of the human survivin gene. PhSurv, human survivin gene promoter; PhSurv(m2) and PhSurv(m4), mutant at Sp1 sites promoters of the human survivin gene; PhSurv269, minimal human survivin gene promoter (269 bp in length); PmSurv, mouse survivin gene promoter; PhTERT, human telomerase reverse transcriptase promoter; LUC, firefly luciferase gene.

Individual promoters used in the study are schematically shown in Fig. 1A.

1. PhSurv. A promoter region of the human survivin (BIRC5) gene (−1456 to +42) [12] which includes all necessary transcription factor binding sites [13]. It is a CG-rich promoter which lacks TATA box and has a number of Sp1 and Sp3 transcription factor binding sites essential for its activity regulation [12], [14].
2. PhSurv269, a shortened promoter region of the human survivin gene (−268 to +1) [12] which retains its promoter activity [12], [15].
3. PmSurv, the minimal promoter region of the mouse survivin gene (−196 to +1) [16] which includes CDE/CHR cell cycle control elements essential for transcription and four widely spaced Sp1 binding sites [16]. This promoter lacks TATA box and has relatively few Sp1/Sp1-like transcription factor binding sites [16].
4. PhTERT, an hTERT −191 to +48 promoter fragment [17], known to be sufficient to drive efficient and specific transcription in hTERT-positive tumor cells [17]. The promoter is also TATA-less and highly GC-rich. A deletion analysis of the PhTERT promoter identified a 181 bp core promoter region upstream of the transcription start site. The core promoter contains several Sp1 sites [17].

The PhSurv and PhTERT promoters include initiator elements and are transcriptionally controlled by a variety of signaling pathways that promote or suppress carcinogenic processes in humans [18], [19]. The activity of both promoters is suppressed in normal cells and initiated due to cancer transformation [18], [19], [20].

The tandem promoter constructs used in the study are shown in Fig. 1B.

### Comparative analysis of the transcriptional activity of PhSurv/PhTERT tandem promoters and their individual components

Transcriptional activity of the promoters was determined in eukaryotic cells by measuring firefly luciferase activity.

As controls, we used non-transfected cells and cells transfected with control plasmids, a promoterless BV-pGL3 plasmid and a PV-pGL3 plasmid containing the reporter firefly luciferase gene under control of an SV40 promoter. Relative activity of the tested promoters was measured on a panel of tumor cell lines of different origin. The cell lines were different not only in origin but also in p53 status, because the activity of the single promoters under study was reported to be dependent on the p53 status of cells [21], [22].

A comparative analysis revealed that the activity of the PhTS and PhST double promoters in most cases exceeded that of both the PhTERT and PhSurv single promoters, whose activity levels were sharply different in five cancer cell lines of the panel (Table 1). The relative activity of the promoters in different cell lines is presented in Table 1. The following conclusions can be made from the data obtained:

1. The activity changes of both single promoters in different cell lines were qualitatively similar, except the Calu-1 cell line, in which the activity of PhSurv was sharply increased. This effect might be due to the lack in the Calu-1 cells of functional p53 protein known to inhibit the activity of PhSurv [22], although other factors can also be involved.
2. The activity of the double promoters in human cell lines was, as a rule, higher than that of their individual components, although slightly lower than the sum of the constituent promoters' activities (except for the A549 cell line) (Table 1).

**Table 1:** Relative activity of the studied single and double promoters in different cell lines.

	Calu-1	A375	A549	PANC-1	HT1080	Average over all cancer cells	IVL-11NS
P53 status	null	wt	wt	mut	wt		wt
PhTERT	0,80±0,1	2,38±0,2	1,68±0,2	0,78±0,1	1,23±0,2	1,37	0,02±0,002
PhSurv	6,31±0,7	1,46±0,2	1,15±0,2	0,38±0,1	0,84±0,2	2,03	0,12±0,014
PhTERT+PhSurv	7,11±0,8	3,84±0,4	2,83±0,4	1,16±0,2	2,07±0,4	3,39	0,14±0,016
**PhST**	**5,08±0,4**	**4,07±0,4**	**2,12±0,2**	**1,44±0,2**	**2,18±0,2**	**2,98**	**0,23±0,004**
**PhTS**	**7,02±0,4**	**3,70±0,2**	**1,17±0,4**	**1,99±0,2**	**0,86±0,1**	**2,95**	**0,06±0,011**
PhSurv269	9,1±1,0	2,20±0,2	6,11±2,7	2,98±0,1	1,08±0,3	4,38	0,19±0,014
PhTERT+PhSurv269	10,85±1,1	4,58±0,4	7,79±2,9	3,76±0,2	2,31±0,3	5,86	0,21±0,016
**PhTSurv269**	**8,80±0,1**	**3,89±0,8**	**10,91±0,9**	**3,96±0,2**	**5,47±1,5**	**6,61**	**0,40±0,034**
PmSurv	5,05±0,7	1,70±0,1	4,62±1,9	2,96±0,1	0,63±0,1	2,99	0,18±0,037
PhTERT+PmSurv	5,85±0,8	4,08±0,3	6,3±2,1	3,74±0,2	1,86±0,3	4,37	0,20±0,039
**PhTmSurv**	**2,88±0,7**	**1,06±0,2**	**1,93±0,9**	**1,38±0,02**	**0,64±0,2**	**1,58**	**0,14±0,008**

The values represent relative promoter activities as ratios of the luciferase activity expressed by plasmids containing promoters under study to the luciferase activity expressed by a pGL3-PV plasmid containing only the SV40 promoter. Mean values (± SEM) of relative luciferase activity were calculated from three independent experiments.

Sign “**+**” in the first column means that data are calculated as an arithmetic sum of activities of single constituent promoters. The average value of promoter activity over five cancer cell lines (Calu-1, A375, A549, PANC-1, and HT1080) was calculated as a sum of the activities in each line divided by 5. The rightmost column corresponds to normal lung fibroblasts IVL-11NS.

The PhTS and PhST double promoters are not active in normal human lung fibroblasts thus retaining tumor specificity characteristic of the single constituent promoters (Table 1). In murine M3 and B16F1 tumor cell lines, both the single and double promoters are practically inactive, which suggests also the retention of species specificity (data not shown).

### Transcription from the PhTS and PhST tandem promoters is initiated only from the proximal promoter, and the number of transcription start sites is higher than in the single promoter

**Figure 2:**
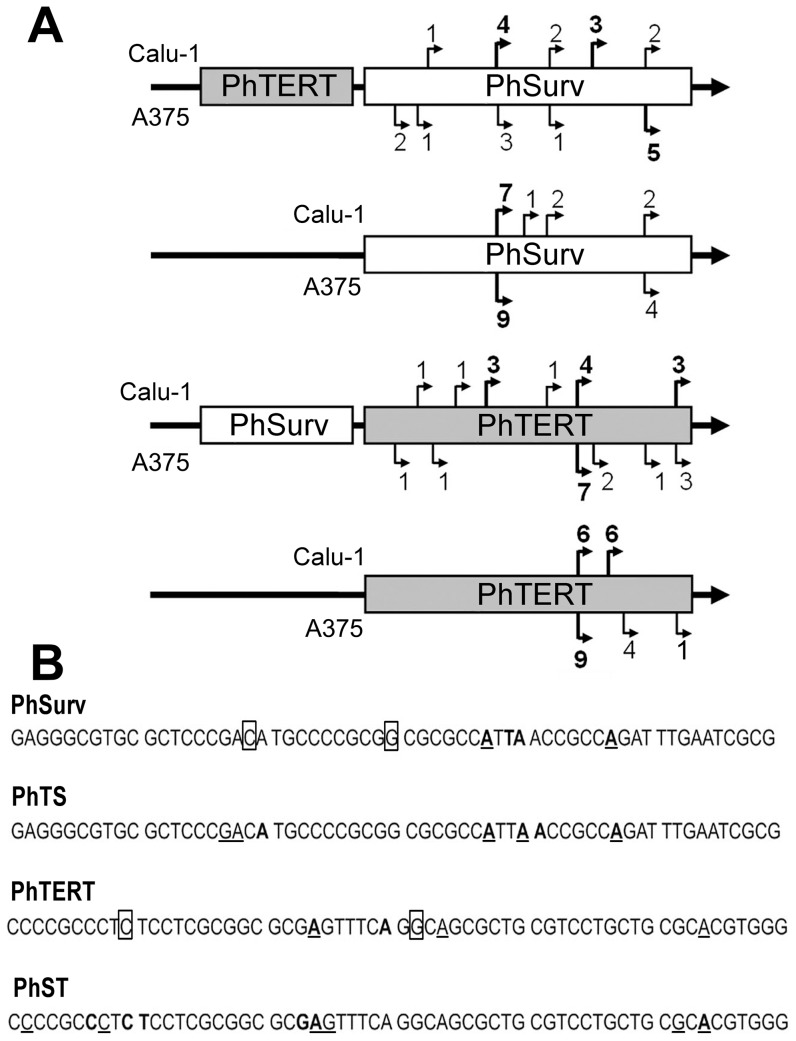
Positions of transcription start sites in single and double promoters in the Calu-1 and A375 cell lines. **A:** Schematic representation of transcription start sites (TSSs) in different promoters under study. Upper and lower arrows mark TSSs in the Calu-1 and A375 cells, respectively. PhSurv, human survivin gene promoter; PhTERT, human telomerase reverse transcriptase promoter. Numbers at the broken arrows represent the number of clones containing the corresponding transcription start site. At least 12 clones were analyzed for each construct. The promoter schemes are out of scale. Actually, all PhTERTs and all the PhSurv promoters have the length of about 240 and 1500 bp, respectively. **B:** Positions of TSSs in single and double promoters in the Calu-1 and A375 cell lines. 60 bp of 3′ promoter regions are shown. TSSs identified in this work for the Calu-1 cell line are shown in bold, and for A375 – underlined; TSSs identified earlier [12], [17], [23] are marked by rectangles.

Fig. 2 demonstrates transcription start sites distribution in the individual and tandem promoters described in the previous section. TSSs were identified using 5′ RLM-RACE analysis of the firefly luciferase gene transcript in the PhSurv-pGL3, PhTERT-pGL3, PhST-pGL3, and PhTS-pGL3 constructs in Calu-1 and A375 cells. These cell lines were chosen because they have different p53 statuses, and the promoter activity in Calu-1 and A375 cells was sharply different (Table 1). The construct with single PhTERT had two TSSs (6 clones for each site) in Calu-1 cell line, and one ‘major’ and two ‘minor’ TSSs (9, 4, and 1 clones for each site, respectively) in A375, consistent with the published data that PhTERT has several TSSs [17], [23]. For PhSurv, we identified one ‘major’ (7 of 12 clones) and three ‘minor’ TSSs (1–2 clones for each site) in Calu-1, and two TSSs (9 and 4 clones for each site) in the A375 cell line. The two single promoters can thus be considered focused [24]. Earlier, two TSSs were identified for the hSurv promoter in HeLa cells [12]. For the hTERT promoter, one major start site was detected in several cell lines [23], and an alternative site was found only in one cell line. The data confirm that these promoters can be characterized as focused. It should be mentioned that positions of TSSs found in our experiments were different in different cell lines (Fig. 2). Tissue specificity of TSSs is a well known phenomenon (see for example [23], [25], [26]).

In contrast, the double promoters PhTS and PhST were characterized by multiple TSSs. We have identified 5 TSSs in PhTS and 6 in PhST in both cell lines (Figs. 2 A, B). The results in Fig. 2 demonstrate that transcription in the tandem constructs was initiated only from the proximal promoter, and the activity of the distal promoter was inhibited. In the case of PhTS, the absence of transcripts initiated from PhTERT might be explained by PCR suppression. PCR amplification of DNA fragments harboring PhSurv could be hampered because of a high GC-content and 1500 bp length of this promoter. However, no transcripts from the distal PhTERT promoter were revealed also by using the 5′RACE approach with primers hSurv_250R and hSurv_150R. It should be noted that although the distal promoters did not initiate transcription they still could modulate the activity of the proximal promoters and cause an appearance of additional transcription start sites.

### Interference between the proximal and distal promoters of the tandems

The data obtained suggest transcriptional interference between the proximal and distal promoters of the tandems. Prior to us, only three head-to-tail tandem whole core promoter constructs were described. Two of them were mentioned in the Introduction section, and the third [27] was a tandem of two pol II promoters derived from the HIV-1 long terminal repeat (LTR). In the first two cases the interference was not reported [10], [11], whereas in the latter case the activity of the proximal promoter was suppressed due to transcription initiated from the distal promoter [27].

Generally, transcriptional interference was described [28], [29], [30], [31] for adjacent transcriptional units but not for head-to-tail fused promoters. It has been observed in various eukaryotic and prokaryotic systems, as well as in viruses [32]. A number of mechanisms have been proposed for transcriptional interference [31]. One of them, promoter competition, could function in tandems with closely spaced promoters. In this case transcriptional interference can be explained by the occupation of one promoter by RNAP that precludes its occupation of the second promoter. Such a mechanism implies that the stronger promoter dominates and functions independently on its position in the tandem. However, we always observed inactivation of only the distal promoter. Therefore, the promoter competition can be apparently ruled out, and the phenomenon observed should be explained by a novel mechanism of promoter interference.

The PhSurv and PhTERT promoters contain seven and five Sp1 transcription factor binding sites, respectively (Fig. 1A) [12], [17], [23]. Sp1 belongs to the zinc finger family of transcription factors and can enhance transcription from a large number of GC-rich promoters in a site-dependent manner [33]. This factor plays a critical role in binding of RNA polymerase II to TSSs in TATA-less promoters and is usually associated with the presence of multiple TSSs [34]. Sp1 binds to its sites as a multimer and is capable of synergic activation of promoters that contain multiple Sp1 binding sites [35], [36], [37]. It has been demonstrated that Sp1 bound to distal regions can interact with Sp1 bound to proximal promoter regions and synergistically activate transcription [36], [38].

Electron microscopy of Sp1/DNA complexes revealed that Sp1 factors formed multimer links (Sp1-bridges) between distant DNA regions thus forming DNA loop structures. It was suggested that transcriptional synergism mediated by Sp1 is due to DNA looping via direct protein-protein association [39].

**Figure 3:**
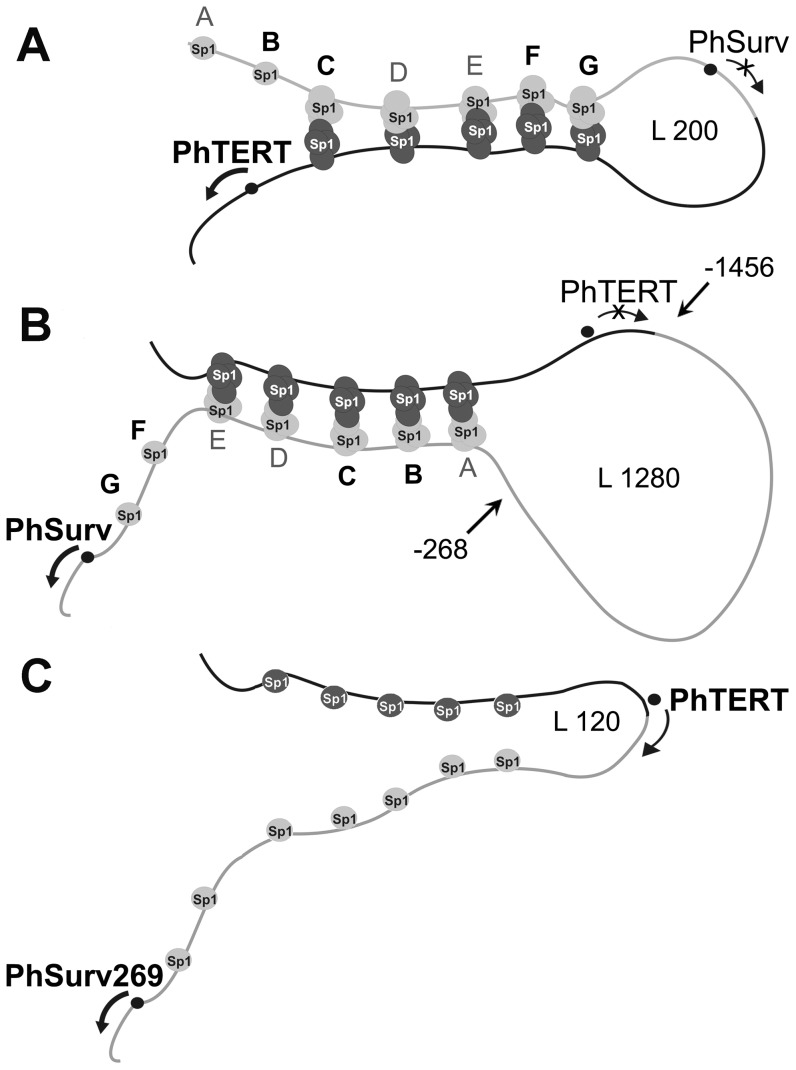
Hypothetic model of distal promoter activity inhibition in double promoters. **A**, **B** and **C** – hypothetic structures of double promoters PhST, PhTS and PhTSurv269, respectively. The constituent promoters PhSurv/PhSurv269 and PhTERT are denoted by grey and black lines, respectively. Small gray and black circles designate Sp1 transcription factors bound to Sp1 sites in the PhSurv/PhSurv269 and PhTERT promoters or in Sp1 multimers. Black dots with arrows indicate transcription start sites. −256 and −1456 in panel **B** delimit part of the PhSurv promoter sequence absent from PhSurv269. L 200, 1280 and 120 (bp) denote the length of the putative loop limited by positions of Sp1 sites. Sp1 sites are marked with letters A–G according to [14]. Black and bold B, C, F and G letters denote the Sp1 sites chosen for mutagenesis. The activity of the distal promoter in the tandem is supposed to be inhibited due to formation of a DNA loop structure with transcription start sites of the distal promoter located inside the loop. In the PhTSurv269 promoter (panel **C**), the length of the putative loop (120 bp) is too small to allow its formation.

In case of the double promoters under study, upstream promoter regions might well be linked to downstream promoter regions via Sp1 multimers to form DNA stem-loop structure, with loops that could include proximal parts of the upstream promoter (Fig. 3 A,B). As topological domains [40], these loops might impose topological constraints on binding of preinitiation complex components with the promoter inside the loop. The formation of open complex is associated with supercoiling of DNA in the vicinity of TSS, which in this model means energetically unfavorable supercoiling of short DNA fragments (Fig. S1). On the other hand, topological constraints do not affect the promoter external to the loop and downstream in our model. The promoter loop structure might also increase the efficiency of transcription initiation from the downstream promoter by bringing additional Sp1 factors closer to this promoter. In the case of PhSurv, a DNA loop can well be formed because its length (1289 bp) is much higher than the persistence length of B form DNA (140–150 bp) [41]. In the case of PhTERT, the hypothetical loop is shorter (200 bp) but still longer than the double-stranded DNA persistence length.

### Comparative analysis of transcriptional activity of the tandem promoters with shortened hypothetical loops

To check the hypothesis above, we have constructed two new tandem promoters – PhTSurv269 and PhTmSurv (Fig. 1B), in which the initial proximal PhSurv was replaced with either (i) a short (269 bp) fragment of the survivin promoter (PhSurv269) that contains six Sp1 sites clustered in a 110-bp segment (as in PhSurv) (Fig. 1A), or (ii) a mouse 198-bp survivin promoter (PmSurv) that contains only four of isolated Sp1 sites within a 160-bp segment (Fig. 1A).

Both promoter fragments are short enough to prevent looping because the length of the hypothetical loop would be as small as ∼120 bp (Fig. 3C), that is considerably smaller than the DNA persistence length.

We estimated the activity of the luciferase gene under transcriptional control of the new tandem promoters in 5 human cell lines, as described above for PhTERT, PhSurv, PhTS and PhST. As can be seen from Table 1, the activity of PhTSurv269 in the Calu-1 and A375 cell lines was similar to that of the tandems containing “long” PhSurv.

To make the quantitative comparison more informative, we calculated the average activity of each promoter in all cancer cell lines tested (Table 1). The values obtained show that the tandem PhTSurv269 has generally the highest activity among the promoters analyzed. In particular, its average initiation level of transcription was 4.8 times higher than that of PhTERT and 3.3 times higher than that of PhSurv. PhTSurv269 also retains cancer-specificity, and its activity in normal lung fibroblasts is low.

### Transcription from “short” tandem promoters is initiated from both proximal and distal promoters

**Figure 4:**
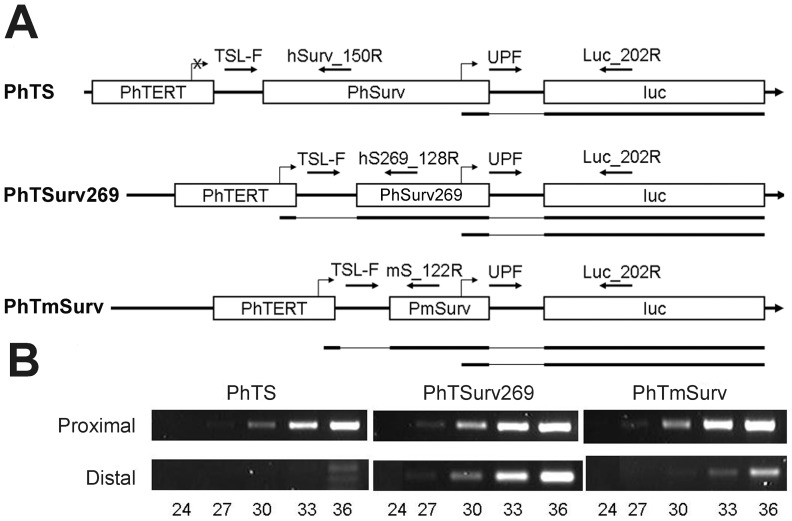
Analysis of transcripts initiated from tandem promoters. **A.** Schemes of double promoter constructs and transcripts initiated from them. Broken arrows indicate transcription start sites; corresponding transcripts are shown below each promoter construct scheme. Thick lines represent sequences of the transcripts complementary to the promoters and luciferase gene, and thin lines correspond to linkers. Horizontal arrows denote PCR primers (for primer sequences see Table 2). The primer pairs TSL-F/hSurv_150R, TSL-F/hS269_128R and TSL-F/mS_122R will amplify transcripts from distal promoters, whereas the pair UPF/Luc_202R – transcripts from both promoters. **B.** RT-PCR amplification of products initiated from distal and proximal promoters. The number of PCR cycles is indicated below each lane. Panel “Distal” represents PCR amplicons of transcripts initiated from the PhTERT promoter within double promoters PhTS, PhTSurv269, and PhTmSurv (primer pairs TSL-F/hSurv_150R, TSL-F/hS269_128R, and TSL-F/mS_122R, respectively). Panel “Proximal” represents RT-PCR amplicons of total transcripts obtained using primers UPF and Luc_202R.

The promoter interference effect in tandem promoter constructs with proximal survivin components was analyzed for PhTS, PhTSurv269 and PhTmSurv using a semi-quantitative RT-PCR technique described earlier [42]. The scheme of the analysis is presented in Fig. 4A. To estimate transcription from the distal PhTERT promoter, we selected a common forward primer TSL-F located in the linker immediately downstream of PhTERT. The reverse primers for each of the survivin promoters (hSurv_150R for the “long” PhSurv promoter, hS269_128R for the short PhSurv269 and mS_122R for PmSurv) were located within the proximal promoters upstream of their TSS sites and at a distance not more than 150 bp from their 5′-ends. The lengths of the PCR amplicons were thus approximately the same in all cases. The PCR results presented in Fig. 4B (panel “Distal”) demonstrate the absence of PCR products in the case of PhTS (with the “long” proximal promoter) and presence of these products in both modified tandems with the shortened proximal promoters.

A similar technique was used to estimate the proximal promoter activity. To this end, a direct UPF primer, located immediately downstream of the proximal promoter and a reverse Luc_202R primer were used. The PCR data are presented in Fig. 4B (panel “Proximal”). As expected, the products were observed for all analyzed constructs.

Thus, the interference effect disappeared on decreasing the proximal promoter length to a size at which the length of hypothetical loops is smaller than the persistence length of double-stranded DNA. This confirms the hypothesis that the activity of the distal promoter is suppressed due to the formation of DNA loops involving parts of this promoter.

### Comparative analysis of the transcriptional activity of tandem promoters containing Sp1 sites-depleted “long” survivin promoters

To further confirm our hypothesis, we have constructed two other tandem promoters, PhSm2T and PhSm4T, with the following mutated survivin promoters in the distal position (Fig. 1):

- a modified human survivin promoter (Sm2) that contains two mutated Sp1 sites (F and G, see Figs. 1 and 3A).
- a modified human survivin promoter (Sm4) that contains four mutated Sp1 sites (B, C, F and G, see Figs. 1 and 3A).

The designation of Sp1 sites and methods of their mutation were described earlier [14]. The Sp1 sites D and E were left intact because of their importance for promoter activity [13], [14].

**Figure 5:**
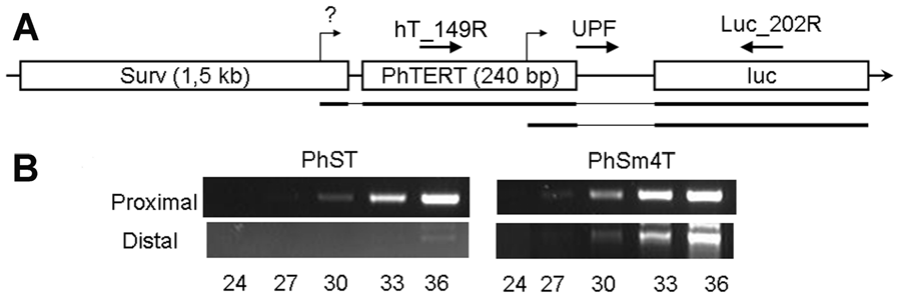
Analysis of transcripts initiated from tandem promoters containing a mutant PhSurv promoter. **A.** Scheme of the double promoter construct and transcripts initiated from it. Surv denotes either non-mutant PhSurv (as part of PhST), or PhSurv(m4) (as part of PhSm4T) with four mutant Sp1 sites. Broken arrows indicate transcription start sites; ? means that the activity of this TSS depends on the Surv promoter used; transcripts are shown below the scheme as straight lines. Thick lines represent sequences of the transcripts complementary to promoters and luciferase gene, and thin lines represent linkers. Horizontal arrows denote PCR primers (for primer sequences see Table 2). The primer pair hT_149R/Luc_202R will amplify transcripts from distal promoters, whereas the pair UPF/Luc_202R – transcripts from both promoters. **B.** RT-PCR amplification of products initiated from distal and proximal promoters. The number of PCR cycles is indicated below each lane. Panel “Distal” represents PCR amplicons of transcripts initiated from survivin promoters with the hT_149R/Luc_202R primer pair. Panel “Proximal” represents RT-PCR amplicons of total transcripts obtained using primers UPF and Luc_202R. PhST and PhSm4T are the names of double promoters.

The promoter interference effect in tandem promoter constructs with distal survivin components was analyzed for PhST, PhSm2T and PhSm4T using a semi-quantitative RT-PCR technique described above. The scheme of the analysis is presented in Fig. 5A. To estimate transcription from the distal survivin promoter, we selected a common forward primer hT_149F located in the PhTERT promoter upstream of its TSSs (see Fig. 2). Luc_202R was used as a reverse primer. The PCR results presented in Fig. 5B (panel “Distal”) demonstrate the absence of PCR products in the case of the PhST promoter and presence of the products with PhSm4T as the distal promoter in which four Sp1 sites were mutated. Thus, the distal promoter of the PhST tandem remained silent.

The activities of the proximal promoter were analyzed as described in the previous chapter. Direct UPF and reverse Luc_202R primers were used. The PCR data are presented in Fig. 5B (panel “Proximal”). As expected, the products were observed for all analyzed constructs.

The disappearance of the interference effect on decreasing the number of Sp1 sites in the quadruplex stem part of the proposed stem-loop structure (Fig. 3) confirms the hypothesis that the distal promoter is inactivated due to its inclusion in a topologically constrained loop structure. Summarizing, the data obtained agree with a stem-loop promoter inactivation mechanism. However, a role of other factors in the suppression effect can not be excluded.

### Conclusion

- In search for universal cancer-specific and strong promoters active in a wide spectrum of cancer types, we spliced the promoters of human telomerase (PhTERT) and human survivin (PhSurv) genes widely used for gene therapy and having the broadest known cancer type spectrum of activity. Two head-to-tail constructs were made: the PhTERT-PhSurv and PhSurv-PhTERT tandems.
- We investigated the transcriptional activity of the tandem promoters formed. Unexpectedly, we discovered that in both constructs only the promoter *proximal* to the transcribed gene retained its ability to initiate transcription, whereas the *distal* promoter remained silent. This kind of promoter interference has never been reported before.
- We put forward a hypothesis explaining a novel mechanism that could operate in head-to-tail positioned closely spaced promoters. The mechanism involves the formation of stem-loop DNA structures (Fig. 3) in which the loop is formed due to binding of multimeric Sp1 transcription factors to their recognition sites located on both promoters of the tandem (Sp1-bridges). The loop in this structure includes at least part of the distal promoter and forms a topological domain [40]. In such a domain untangling of two DNA strands necessary for transcription initiation is topologically constrained. Thus, inhibition of the transcription from the distal promoter is not due to promoter competition, but to topological constraints imposed by looping of the distal promoter.
- The hypothesized mechanism was confirmed by shortening the loop length to make the loop formation energetically unfavorable, and by removing Sp1 sites from the promoter tandems to hamper the loop formation. In both cases the activity of the distal promoter was restored in accordance with the hypothesis.
- Based on the data obtained, the design of new tandem promoters should take into account possible undesirable looping of the constructs. To prevent the looping, we can recommend to avoid too long constructs and choose constituent promoters with a low number of multimeric transcription factor binding sites.
- Finally, we found a novel and efficient tandem promoter combination PhTERT-PhSurv269 with a shortened hSurv promoter. This tandem had the highest and more uniform promoter expression level in different cancer cells among all cancer specific promoters tested. In particular, its average expression level was 4.8 times higher than that of the widely used in gene therapy PhTERT and 3.3 times higher than that of another cancer-specific promoter PhSurv. The tandem also retained cancer-specificity.

We hope, that the data obtained would be useful both to researchers looking for new promoter constructs for genetic therapy and to those who study mechanisms of transcription initiation.

## Materials and Methods

### Cell lines

Cancer cell lines A549 (lung carcinoma), HT1080 (fibrosarcoma), PANC-1 (carcinoma of the exocrine pancreas), A375 (human melanoma), B16F1 (murine melanoma), M3 (Claudman mouse melanoma) were obtained from American Type Culture Collection (ATCC, Manassas, VA). The human lung cancer cell line Calu-1 (epidermoid lung carcinoma) was obtained from European Collection of Cell Cultures (ECACC, Salisbury, UK). Fibroblasts IVL-11NS (NLF) were obtained from normal lung tissue adjacent to tumor according to a standard protocol [43]. The specimen was obtained from a lung tumor surgery patient at the Vishnevsky Institute of Surgery (Moscow, Russia), as described previously [44]. The cells were grown in DMEM/F12 (1∶1) medium containing 10% fetal calf serum, 60 µg/ml penicillin, 100 µg/ml streptomycin, and 0.25 µg/ml amphotericin (Invitrogen, USA) at 37°C and 5% CO2.

### Patients and tissue specimens

A normal lung fibroblast cell line IVL-11NS (NLF) was provided by the Vishnevsky Institute of Surgery. A surgical tumor specimen for establishing this line was obtained from a patient with diagnosed lung cancer who has undergone complete resection of tumor at the Vishnevsky Institute of Surgery. The final diagnosis was confirmed by hematoxylin-eosin staining of paraffin blocks after the operation. The patient did not receive chemo- or radiotherapy before surgery. The sample was obtained with the verbal consent of the patient. The project protocol was approved by the Institutional Review Board at the Vishnevsky Institute of Surgery.

### Construction of expression vectors

A PhSurv-pGL3 plasmid with a single PhSurv promoter was obtained earlier [13]. A PhTERT-pGL3 construct was kindly provided by Dr. Korobko (Institute of Gene Biology RAS, Moscow). A PhSurv269-pGL3 construct was kindly provided by Dr. Kostina (Shemyakin and Ovchinnikov Institute of Bioorganic Chemistry RAS, Moscow). A PmSurv-pGL3 plasmid with a single PmSurv promoter was obtained earlier in our laboratory. The promoter fragment mSurv was amplified on a template of mouse genomic DNA with primers 5′-AGATCTCCACGCCCACAAGGCCAGGC-3′ and 5′-AAGCTTATGATGGCGTCACCACAACC-3′ that contained *Bgl*II и *Hin*dIII sites. The amplified mSurv was cloned into the pGEM-T vector, sequenced and then cloned between *Bgl*II and *Hin*dIII sites into the pGL3-Basic vector. To construct double promoters PhST and PhTS, a 1498 bp survivin promoter DNA was obtained by hydrolysis of the PhSurv-pGL3 plasmid with BglII and HindIII restriction enzymes and then blunt ended with the Klenow fragment. The DNA fragment obtained was ligated to the PhTERT-pGL3 vector pre-linearized by cleavage with HindIII or KpnI and blunt ended with the Klenow fragment. To obtain the PhTSurv269 promoter, a 269 bp human survivin promoter DNA was isolated by hydrolysis of the PhSurv269-pGL3 plasmid with BglII and HindIII restriction enzymes, and then blunt ended with the Klenow fragment. The DNA fragment obtained was ligated to the PhTERT-pGL3 vector pre-linearized by cleavage with HindIII and blunt ended with the Klenow fragment. To obtain the PhTmSurv promoter, a 196 bp mouse survivin promoter DNA was isolated by hydrolysis of the PhSurv269-pGL3 plasmid with BglII and HindIII restriction enzymes and blunt ended with the Klenow fragment. The DNA fragment obtained was ligated to the PhTERT-pGL3 vector pre-linearized by cleavage with HindIII and blunt ended with the Klenow fragment. As a result, we obtained PhTS-pGL3, PhST-pGL3, PhTSurv269-pGL3 and PhTmSurv-pGL3 vectors with the firefly luciferase gene under control of several tandem combinations of single promoters upstream of the luciferase gene start codon (Fig. 1B). The structure of all the constructs obtained was confirmed by sequencing.

### Site-directed mutagenesis and construction of tandem promoters containing mutated survivin promoters

To mutate two Sp1 sites in the “long” surviving promoter, we hydrolyzed the PhSurv-pGL3 plasmid with SacII restriction enzyme to excise a 46 bp fragment containing two Sp1 sites (F and G, see Fig. 3A). Using long primers FG-For and FG-Rev, we generated a new artificial 46 bp duplex with mutated Sp1 sites and then integrated this duplex into the previously cleaved plasmid. As a result, we obtained PhSurv(m2)-pGL3 vector with two mutated Sp1 sites. To mutate four Sp1 sites, we used a method of mutagenesis by overlap extension (described in [45]). To this end, we used PhSurv(m2)-pGL3 vector as a template and Pfu DNA Polymerase (Fermentas, Canada) with primer pairs PstI/S-For and MutBC-Rev to synthesize a 715 bp fragment, and primer pairs Hind/S-Rev and MutBC-For to synthesize a 161 bp fragment. Then we used primer pairs PstI/S-For and Hind/S-Rev to generate a combined 852 bp PCR fragment from previously obtained short fragments. This fragment was hydrolyzed with PstI and HindIII restriction enzymes and then ligated to the PhSurv-pGL3 plasmid pre-hydrolyzed with the same restriction enzymes. As a result, we obtained PhSurv(m4)-pGL3 vector with four mutated Sp1 sites (B, C, F and G, see Fig. 3A). The obtained plasmids were used to prepare double promoters PhSurv(m2)-PhTERT (PhSm2T) and PhSurv(m4)-PhTERT (PhSm4T) as described above for PhST. The structure of all the constructs obtained was confirmed by sequencing.

### Transfection of cells

Cells were transfected in 24-well plates using Lipofectamine 2000 (Invitrogen) according to the manufacturer's recommendations. Transfection was done with 0.88 µg mixture of a reporter plasmid carrying the firefly luciferase gene and an internal control plasmid pRL-TK (Promega) in the molar ratio of 10∶1. In 48 h after transfection, the activity of firefly and *Renilla reniformis* luciferases was measured in cell extracts using a Dual-Luciferase Reporter Assay System (Promega) and a GENios Pro (Tecan, Switzerland) luminometer. In parallel experiments, cells were transfected with a promoterless BV-pGL3 plasmid or a PV-pGL3 plasmid, containing only the SV40 promoter (positive control). For each construct under study, at least three independent transfections were performed.

The values in Table 1 represent relative promoter activities as a ratio of the luciferase activity in extracts of cells transfected with plasmids containing promoters under study to the activity in extracts of cells transfected with plasmid containing the SV40 promoter (pGL3-PV). Mean values (± SEM) of the relative luciferase activity were calculated from three independent experiments using Microsoft Office Excel program.

### Location of transcription start sites in the constructs obtained

Calu-1 and A375 cells were transfected with the PhSurv-pGL3, PhTERT-pGL3, PhST-pGL3, or PhTS-pGL3 constructs. In 48 h after transfection, the cells were harvested, total RNA was isolated with a RNeasy Mini Kit (Qiagen, USA) according to the manufacturer's protocols. To locate transcription start sites, we used a FirstChoice RLM-RACE Kit (Ambion, USA) following the manufacturer's recommendations. For nested PCR, we used gene-specific primers Luc_385R and Luc_202R, hSurv_250R, hSurv_150R (Table 2). The reaction products were cloned into a pAL-TA vector (Evrogen, Russia), and sequenced. For all four constructs (PhSurv, PhTERT, PhTS, and PhST), at least 12 resulting clones were sequenced.

**Table 2:** Primers used in the experiments.

Primer	Primer sequence, 5′–3′	Annealing temperature
Luc_385R	AAACGAACACCACGGTAGGCT	60
Luc_202R	TCATAGCTTCTGCCAACCGAAC	60
hSurv_250R	AGTAGCTGAGATTAAAGGCATGCA	60
hSurv_150R	TCCTGACCTCAAGTGATCTGCCT	60
TSL-F	TGATCTGCGATCTAAGTAAG	56
hS269_128R	CGGGGTGTGCCGGGAGT	56
mS_122R	CAGAGCATGCCGGGAGAG	56
UPF	CTTGGCATTCCGGTACTGT	56
FG-For	GGGGGGTGGACCAACTAAGAGGGAAAG CGCTCCCGACATGCCCCGC	-
FG-Rev	GGGGCATGTCGGGAGCGCTTTCCCTCT TAGTTGGTCCACCCCCCGC	-
PstI/S-For	TACTGCAGGACTTACTGTTGGT	56
MutBC-Rev	AATGCGTGGCTCTAACAGTGGTCGCGGT	56
MutBC-For	TGTTAGAGCCACGCATTGGGAGGACTACA	56
Hind/S-Rev	TCCAAGCTTCGCGATTCAAATCT	56
hT_149F	GGGCCCTCCCAGCCCCT	60

### RNA isolation and RT-PCR

Total RNA from transfected cell lines isolated as described above was further treated with DNase I (Qiagen) to remove residual DNA. cDNA synthesis was performed according to the manufacturer's protocol using random hexamer primers (Perkin Elmer), with (+RT) or without (−RT) addition of PowerScript II reverse transcriptase (Clontech).

A cDNA equivalent of 20 ng total RNA was used as template in each PCR, and the amplification was carried out using specific primers as described earlier [42]. PCR primer sequences are presented in Table 2.

In parallel, control tests for purity of PCR reaction mixtures and non-amplification of genomic and plasmid DNA were performed. All RT-PCR reactions were reproduced at least three times in independent experiments.

## Supporting Information

Figure S1 **Model of initiation complex loop structure formed on a double promoter through Sp1 interaction.** Grey circles denote Sp1 proteins associated with Sp1 binding sites of promoters. Due to a small size of the formed loop, the supercoiling in this system is energetically unfavorable.
(TIF)
