# Correction: Activity of the Upstream Component of Tandem TERT/Survivin Promoters Depends on Features of the Downstream Component

**DOI:** 10.1371/annotation/9fbcbbe8-8155-4611-9de1-8708de029017

**Published:** 2013-07-18

**Authors:** Irina V. Alekseenko, Victor V. Pleshkan, Eugene P. Kopantzev, Elena A. Stukacheva, Igor P. Chernov, Tatyana V. Vinogradova, Eugene D. Sverdlov

There is an error in the Funding Statement. The correct Funding Statement is the following:

This work was supported by Presidential grant for Scientific Schools (5638.2010.4) and grant from the Molecular and Cellular Biology Program of the Presidium of the Russian Academy of Sciences. The funders had no role in study design, data collection and analysis, decision to publish, or preparation of the manuscript.

The following statement is missing from the Acknowledgments:

The authors are grateful to Dr. M. Pikunov (Vishnevsky Institute of Surgery, Moscow, Russia) for providing us with fibroblasts IVL 11NS (NLF), to Dr. V. Korobko (Institute of Gene Biology RAS, Moscow) for providing us a PhTERT-pGL3 construct and to Dr. M. Zinovyeva for aid in preparation of RNA and cDNA samples. 

