# Supplementary figures and images for: Activity of the Upstream Component of Tandem TERT/Survivin Promoters Depends on Features of the Downstream Component
Source: PLoS One. 2012 Oct 3;7(10):e46474. doi: 10.1371/journal.pone.0046474 (PMC3463601; doi:10.1371/journal.pone.0046474)

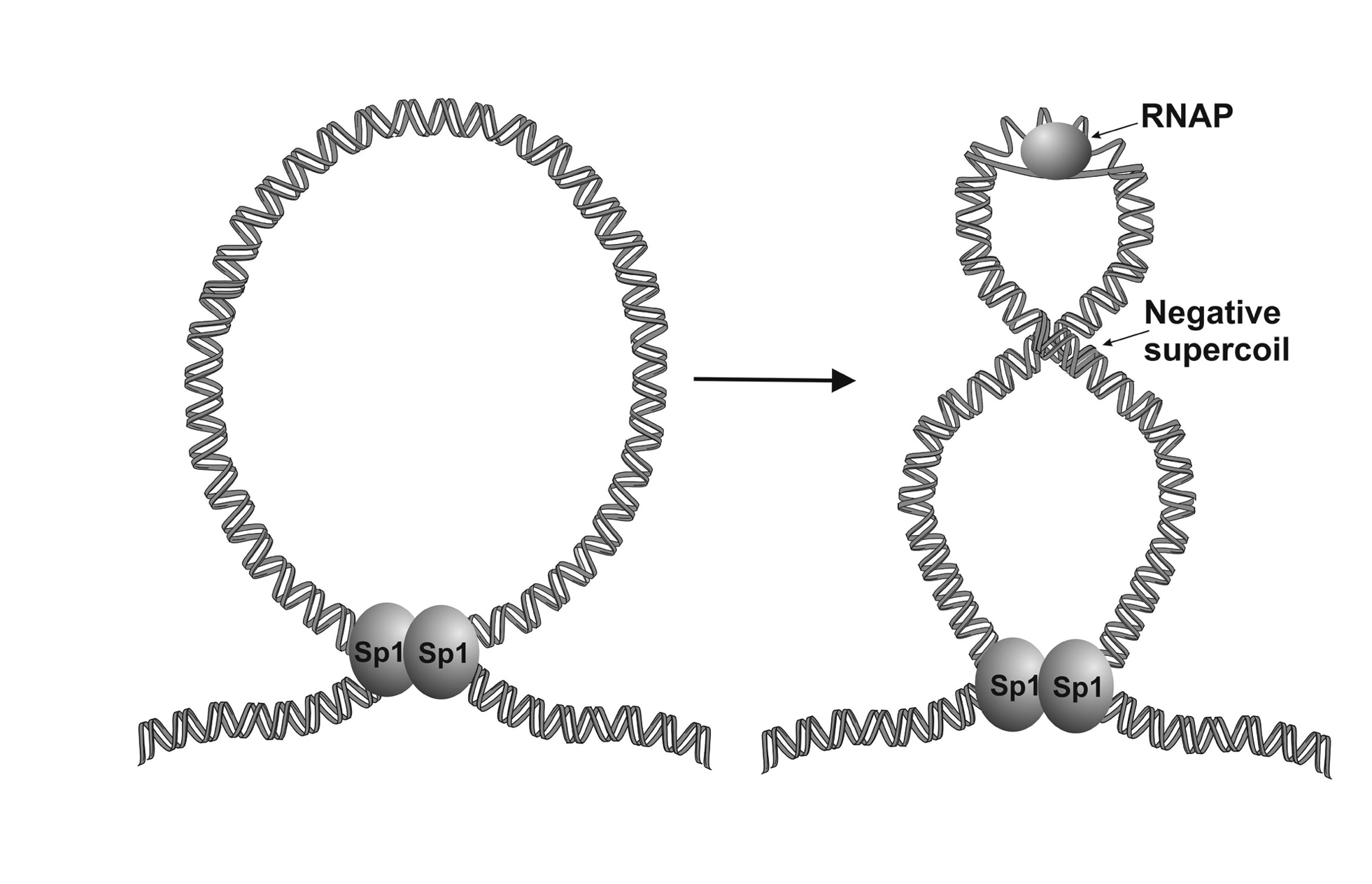

Supplement: Figure S1 — Model of initiation complex loop structure formed on a double promoter through Sp1 interaction. Grey circles denote Sp1 proteins associated with Sp1 binding sites of promoters. Due to a small size of the formed loop, the supercoiling in this system is energetically unfavorable. (TIF) [file pone.0046474.s001.tif]
